# Versatile Action of Picomolar Gradients of Progesterone on Different Sperm Subpopulations

**DOI:** 10.1371/journal.pone.0091181

**Published:** 2014-03-10

**Authors:** Diego Rafael Uñates, Héctor Alejandro Guidobaldi, Laura Virginia Gatica, Marisa Angélica Cubilla, María Eugenia Teves, Ayelén Moreno, Laura Cecilia Giojalas

**Affiliations:** Centro de Biología Celular y Molecular (UNC) and Instituto de Investigaciones Biológicas y Tecnológicas (UNC-CONICET), Córdoba, Argentina; Universidad Nacional Autónoma de México, Mexico

## Abstract

High step concentrations of progesterone may stimulate various sperm physiological processes, such as priming and the acrosome reaction. However, approaching the egg, spermatozoa face increasing concentrations of the hormone, as it is secreted by the cumulus cells and then passively diffuses along the cumulus matrix and beyond. In this context, several questions arise: are spermatozoa sensitive to the steroid gradients as they undergo priming and the acrosome reaction? If so, what are the functional gradual concentrations of progesterone? Do spermatozoa in different physiological states respond differentially to steroid gradients? To answer these questions, spermatozoa were confronted with progesterone gradients generated by different hormone concentrations (1 pM to 100 µM). Brief exposure to a 10 pM progesterone gradient stimulated priming for the acrosome reaction in one sperm subpopulation, and simultaneously induced the acrosome reaction in a different sperm subpopulation. This effect was not observed in non-capacitated cells or when progesterone was homogeneously distributed. The results suggest a versatile role of the gradual distribution of very low doses of progesterone, which selectively stimulate the priming and the acrosome reaction in different sperm subpopulations.

## Introduction

In biological organisms, molecules diffuse from the source, generating molecular gradients which modulate important cellular functions such as growth, differentiation and migration [1]. Progesterone, a highly phylogenetically-conserved biomolecule with a variety of functions in reproduction, may also be distributed along a gradient. For example, by the time of ovulation it is secreted by the cumulus cells that surround the oocyte [2], [3]. These cells are progressively distributed along the cumulus matrix, closer to each other near the oocyte and becoming sparse towards the cumulus periphery [4]. Thus, upon secretion, progesterone diffuses forming a concentration gradient along the cumulus and beyond [5]. In this context, it is reasonable to infer that spermatozoa may face a gradual increase of progesterone concentration during their approach to the egg complex, but are spermatozoa sensitive to steroid gradients?

Even though progesterone stimulates a myriad of sperm functions [6], almost all the studies have been done with a homogeneous distribution of the hormone. The reports showing that human spermatozoa are able to sense progesterone gradients are mainly related to sperm chemotaxis, a guiding mechanism that is exclusively dependent on the gradual distribution of the attractant molecule [7]. In this way, spermatozoa can chemotactically orient their movement towards the source of very low doses of progesterone [5], [8]–[12].

On the other hand, each spermatozoon asynchronously experiences sequential physiological changes throughout its lifespan, giving rise to a sperm population that is naturally heterogeneous. Such physiological changes are mostly related to sperm capacitation, a cellular state that is necessary for fertilizing the egg. Capacitation is a transient and unique event in sperm life, lasting 1–4 hours; afterwards, the spermatozoon becomes irreversibly postcapacitated [13]. It was suggested that, at any given time, the sperm sample contains some sperm that are in progress to capacitation, while others are either already capacitated or postcapacitated [14]. So it is not surprising that the sperm response to a biomolecular gradient is dependent on the sperm's physiological state. Indeed, spermatozoa must be capacitated to sense a chemoattractant gradient [12], [13], [15].

One of the consequences of being capacitated is that the spermatozoon is able to further undergo the induced acrosome reaction, a requirement for a successful fertilization [16]. During the last steps of capacitation the sperm cells are prepared for the induced acrosome reaction (named “priming”, [17], [18]). For years, the zona pellucida has been considered the physiological stimulus for the acrosome reaction under *in vivo* conditions [19]. However, it was recently reported in mouse that spermatozoa that fertilize the egg undergo the acrosome reaction far from the zona pellucida [20], [21], opening up the possibility of another physiological inducer for the acrosomal exocytosis. Progesterone has been reported to stimulate both priming and the acrosome reaction; however, those studies were performed with homogeneous distribution of micromolar doses of the hormone [6]. In contrast, we recently observed that a brief exposure to picomolar gradients of progesterone stimulates or completes sperm capacitation [12].

Within this context, we hypothesized that, under natural conditions, the acrosome reaction and priming for the acrosome reaction are stimulated by concentration gradients of progesterone affecting spermatozoa according to their physiological state. Our findings suggest a versatile role of the gradual distribution of very low doses of progesterone, which selectively stimulate the priming and the acrosome reaction in different sperm subpopulations.

## Materials and Methods

### Ethics statement

Experiments were designed for human semen samples in accordance with the Declaration of Helsinki. The study was conducted with the approval of the Ethics Committee of the Hospital Nacional de Clínicas (Universidad Nacional de Córdoba, Argentina). All participants gave written informed consent.

### Sperm preparation

The semen samples were obtained from healthy donors after 3–5 days of abstinence. Only those samples exhibiting normal seminal parameters according to the WHO criteria [22] were included in the study. Spermatozoa were separated from the seminal plasma using a discontinuous Percoll gradient [8] (Sigma- Aldrich, St. Louis, USA) in HAM F-10 medium with L-glutamine and 25 mM Hepes (Gibco, New York, USA). Then, the highly motile sperm population was adjusted to 8×10^6^ cells/ml in HAM F-10 supplemented with 1% human serum albumin (Laboratorio de Hemoderivados, Universidad Nacional de Córdoba, Argentina) and incubated at 37°C in 5% CO_2_ on air for 4 h. All the experiments were carried out with cells incubated under the above capacitating conditions unless otherwise specified.

### Systems to generate gradients of progesterone

**Figure 1:**
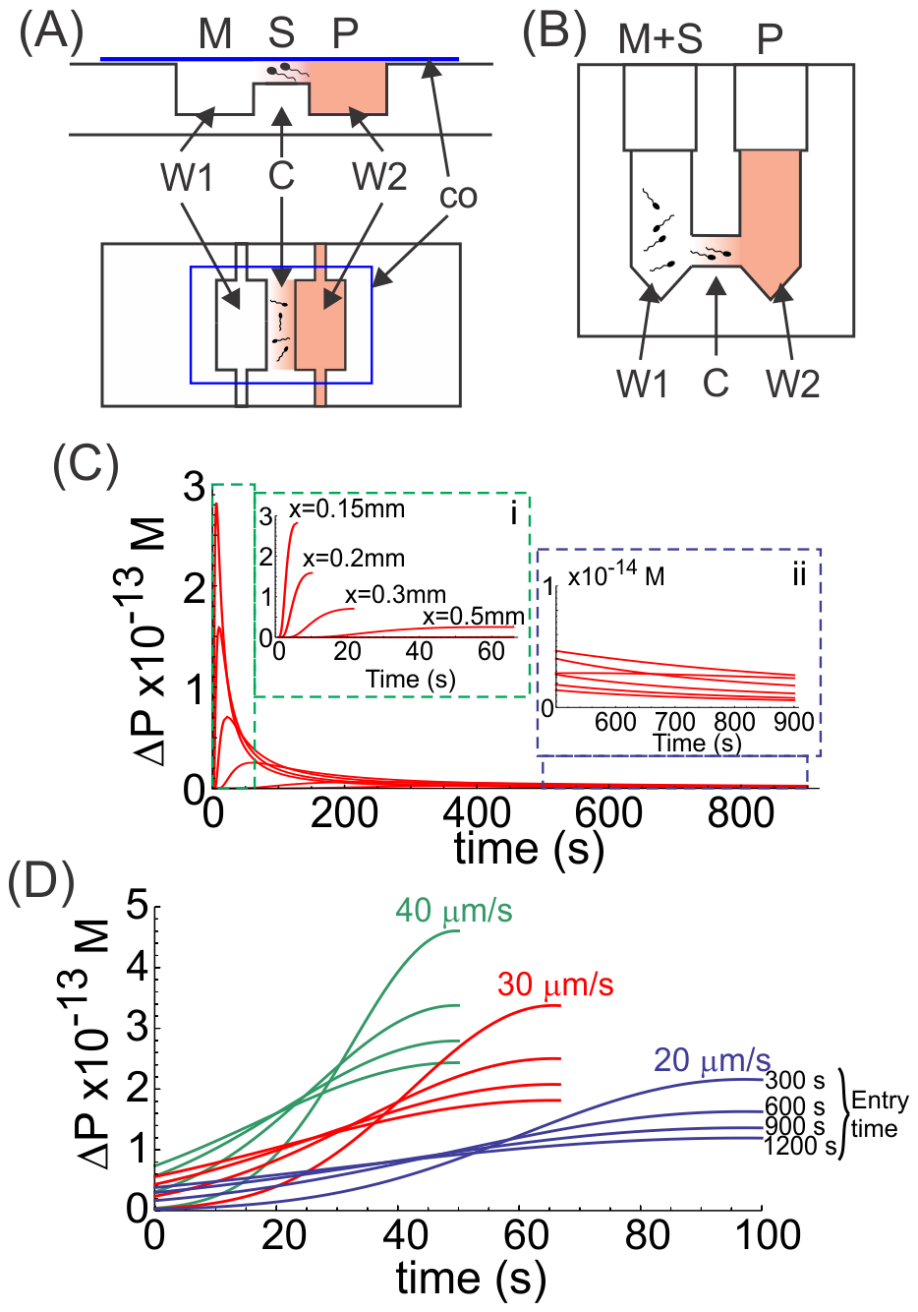
Chambers for generating progesterone gradients. Both chambers consist of two wells (W1 and W2) with a connection (C) between them, where progesterone (P) is loaded in W2 and diffuses to W1 (containing culture medium; M) through the connection, forming a one-dimensional concentration gradient. The difference between systems lies in the way spermatozoa (S) are exposed to the gradient. A, in the CH chamber the sperm population is stuck to a coverslip (co) which is positioned upside down at the connection containing the progesterone gradient; therefore, the cells are exposed to the progesterone gradient from a fixed position. B, in the SSA device, spermatozoa are loaded in W1, facing the progesterone gradient while swimming across the connection between wells. C and D, theoretical representation of the rate of change of progesterone (ΔP) over time in the CH and SSA chambers, respectively. ΔP was calculated according to the formula [P]_t+1_ - [P]_t_, where [P] is the concentration of progesterone and *t* is the time. C, ΔP shown in cells positioned at different arbitrary distance (x) from the progesterone-containing well (the closest position being 0.15 mm); insets: i, represents a magnification of ΔP values during the first 60 s of the experiment, in cells located at different distances from the progesterone source; ii, represents a magnification of ΔP for the same cells shown in i, but from 500 s until the end of the experiment. D, ΔP is shown in 3 theoretical cells swimming at different linear speeds (20, 30 and 40 µm/s) getting into the connection between wells at different times (300 s up to 1200 s).

*a) Chemotaxis chamber* (*CH*; Fig. 1A). Spermatozoa were adhered to a coverslip according to the following procedure. Briefly, two parallel lines were drawn in the middle of the upper side of a coverslip of a width equivalent to that of the connection between wells. Then, on the obverse of this strip, several drops of 2 µl of 0.1% Poly-L-lysine (Biochrom, Cambridge, United Kingdom) in distilled water were spread. Once the Poly-L-lysine was dry, 100 µl of the sperm suspension at 8×10^6^ sperm/ml were carefully distributed over it. After 5 min, the coverslip was washed with culture medium to remove the non-adhered spermatozoa. This procedure yielded a median of 60 cells per field, adhered by their heads, of which 87±1% were alive (as verified by Hoechst vital staining) and 66±3% had moving tails (as verified under the light microscope). The coverslip with adhered cells was used to prepare the CH chamber as follows. Drops of culture medium were placed over the connection between both wells and the coverslip was placed upside down with the parallel strips coinciding with the borders of the connection of the chamber. The sides of the coverslip were first adhered to the chamber surface with vaseline and the borders were sealed with wax. Then, one well (W1) was filled with culture medium through the lateral openings which were immediately closed with wax. The same procedure was followed to place the solution of progesterone (10 pM; Sigma-Aldrich, St. Louis, USA) in the other well (W2). In this way both wells are communicated by the connection in which the gradient is formed by simple diffusion from W2 to W1, by which the cells are gradually exposed to progesterone from a fixed position in a hermetic system. The chamber was incubated at 37°C for 15 min. As a negative control, a chamber without a progesterone gradient along the connection was prepared, with either culture medium or a homogeneous concentration of progesterone placed in both wells and over the connection. *b) Sperm Selection Assay chamber* (*SSA*; Fig. 1B). The connection between wells was filled with culture medium and a cap of W2 was placed over it. Then, a suspension with 6×10^6^ sperm/ml was placed in W1, which was closed with a cap. Immediately, the cap of W2 was removed and the progesterone solution was added to the well, closing it with the cap. The device was incubated at 37°C in a 5% CO_2_ on air for 20 min. The progesterone gradient is also formed by simple diffusion, and the spermatozoa are gradually exposed to progesterone while swimming across the connection from W1 to W2. The cap of W2 was taken off and the sperm suspension was removed from the well and the same procedure was followed with the cells in W1. As a negative control, culture medium was placed in W2.

### Determination of the percentage of acrosome-reacted spermatozoa

The acrosome staining was performed in the coverslip recovered from the CH chamber containing the cells or in the slide where sperm recovered from the SSA were placed. The acrosome was labeled with Coomassie Blue G 250 (Fluka Analytical, USA) according to Larson and Miller [23], which clearly distinguishes the different states of the acrosome (intact or reacted) and does not stain the Poly-L-lysine substrate. Briefly, spermatozoa were first fixed with 1% formaldehyde for 20 min at room temperature. Then, the cells were washed 3 times with 100 mM ammonium acetate for 7 min each at 300×g. Spermatozoa were stained for 10 min with 0.22% Coomassie Blue in a solution of 10% acetic acid and 50% methanol. After washing with distilled water, the cell preparations were let to dry and mounted with 90% glycerol in PBS. The acrosome state was observed under light microscope at 1,000×, and two patterns were identified: dark blue and light blue acrosome, representative of intact and reacted acrosome, respectively. The state of the acrosome was evaluated in 200–600 cells and the percentage of acrosome-reacted sperm was determined by dividing the number of sperm with the acrosome reacted by the total number of cells counted per 100. To evaluate whether progesterone induced per se the acrosome reaction, the percentage of acrosome-reacted sperm was statistically compared to sperm not exposed to progesterone. To evaluate the priming effect of progesterone, the level of sperm that underwent the acrosome reaction induced by calcium ionophore was determined as the difference between the percentages of acrosome-reacted sperm with and without ionophore treatment. Progesterone and A23187 calcium ionophore (Sigma- Aldrich, St. Louis, USA) dilutions were prepared in culture medium from a 1 mM and 2 mM stock solution in DMSO (Sigma- Aldrich, St. Louis, USA), respectively. Serial dilutions of progesterone were prepared and used only once. Calcium ionophore was added at a final concentration of 8 µM for 30 min at 37°C.

### Intracellular calcium determination

Sperm suspension at 7×10^6^ sperm/ml was loaded with 1 µM Fluo 4 AM (Molecular Probes; Paisley, UK), diluted in culture medium from a stock solution of 1 mM in DMSO for 30 min at 37°C. Samples were then centrifuged with culture medium for 7 min at 300×g. Spermatozoa were adhered to a coverslip as described above, which was used in two different devices under gradual (CH chamber) or homogeneous (imaging chamber) distribution of progesterone. The CH chamber was assembled as described above and the recording for calcium signaling started 1 minute after closing the chamber, lasting for 15 min at a frequency of 1 frame each 5 seconds (0.2 Hz), under an exposure time of 600 ms. For preparing the imaging chamber, the coverslip containing the cells was placed at the bottom of the chamber and the progesterone solution was added from above. The recording of calcium signaling started 1 minute before the addition of progesterone and continued for 7 additional minutes, at the same frequency and exposure time than the CH chamber. Calcium imaging was performed at room temperature in an inverted fluorescence microscope (Nikon Ti-S/L100) coupled with a digital camera (Nikon DS-Qi1Mc) where the fluorescence illumination was supplied by LED stroboscopic light, controlled by NIS Elements software (Nikon, USA), minimizing light toxicity [24]. Cells showing a fluorescence head were considered live and only these were analyzed (dead or dying cells do not retain the dye; [25], [26]). The images were captured with the Nis Elements software (Nikon, USA). In the step experiment, the time recorded before progesterone application was considered as a negative control. In the gradient experiment, a chamber without progesterone was run as a negative control, which was discounted to the corresponding progesterone data. The calcium fluorescence intensity was analyzed with the ImageJ software (ver. 1.46, NIH, USA) in at least 50 cells per treatment. The fluorescence intensity variations were analyzed with the Microsoft Excel software, and expressed as the difference in fluorescence intensity according to the formula: Δ% Fluorescence = (F_1_/F_0_×100)-100, where F_1_ is the fluorescence intensity at any time point and F_0_ is the initial fluorescence intensity recorded.

### Statistical analysis

At least 3 independent experiments were performed with semen samples from different donors, analyzing in each treatment 200-600 sperm for acrosome reaction and 50 sperm for calcium measurements. Statistical differences between treatments were determined by means of one-way ANOVA and the Tukey-Kramer tests with the SigmaStat software (SPSS, Inc, USA).

## Results

### Experimental design

The progesterone gradients were generated in two types of devices: the chemotaxis chamber, which was previously used to study sperm chemotactic behavior by videomicroscopy and image analysis (CH; Fig. 1A; [10], [15]), and the Sperm Selection Assay chamber, which was developed by us to recruit capacitated sperm by chemotaxis towards progesterone (SSA; Fig. 1B; [12]). Both chambers are similar, consisting of two interconnected wells (see further description in Materials and Methods section). In each case, progesterone is asymmetrically loaded in one well and immediately diffuses through the connection, forming a one-dimensional concentration gradient. The length of the connection is equivalent in both systems, which, combined with a defined procedure of use, guarantees the formation of the progesterone gradient, while the steroid concentration along the connection and the time can be estimated by Ficks' law for simple diffusion [12]. The difference between systems lies in the way spermatozoa are exposed to the gradient. In the CH chamber, the sperm population is stuck to a coverslip, thus exposing the cells to the progesterone gradient from a fixed position. In the SSA device, spermatozoa are loaded in the well opposite to the progesterone well and are thus exposed to the progesterone gradient while swimming across the connection. In the CH system, two chambers were run in parallel and, after exposure to progesterone, the contents of both wells were replaced with either calcium ionophore or culture medium. Then the coverslip was removed and the cells stained to visualize the acrosome state. In the SSA chamber, at the end of the exposure to the progesterone gradient, the sperm suspension removed from both wells was divided in two aliquots, each stimulated or not with calcium ionophore and then the cells were stained to visualize the acrosome state. Thus, the effect of progesterone was evaluated by determining the level of acrosome-reacted sperm in the population stuck to the coverslip (CH chamber) and accumulated in both wells (SSA chamber), as described in Materials and Methods.

### Interaction of spermatozoa with the pM progesterone gradient inside the CH and SSA chambers

In the CH chamber where spermatozoa are stuck along the connection between wells, the cells sense the change in the progesterone concentration (ΔP) over time, which may vary according to their position in relation to the source of progesterone. Thus, ΔP rapidly increases during the first 100 s, but the magnitude of the peak will depend on the sperm's position along the connection between wells (Fig. 1 C and inset i). Then, a decrease in ΔP is observed in which the higher the ΔP peak (meaning closer to the progesterone source) the faster the decrease in the ΔP (Fig. 1 C). After approximately 600 s of exposure to the progesterone gradient, the cells located at any position are exposed to similar constant values of ΔP (Fig. 1 C and inset ii). In the case of the SSA chamber, spermatozoa may swim into the connection between wells at different times during the experiment and at different speeds since the sperm population is heterogeneous, though the movement is progressively linear, as expected for a 10 pM gradient of progesterone [5], [12]. When a sperm cell gets into the connection early during the experiment, it will sense increasing ΔP, reaching the higher ΔP value at the end of the connection tube (Fig 1 D; e.g., entry time 300 s). Conversely, a sperm cell entering the connection later will experience a smoother ΔP variation with a smaller ΔP value at the end of the tube compared with an earlier sperm (Fig. 1 D; e.g., entry time 1200 s). Thus, the sperm speed and the time of getting into the connection tube influence the magnitude of ΔP sensed. However, independently of the sperm speed, at the end of the experimental period (1200 s) the sperm cells sense a relatively constant ΔP. Comparing both systems, a spermatozoon that gets into the connection of the SSA chamber early may sense a similar ΔP to one positioned in the CH chamber near the progesterone well. Conversely, a sperm cell entering the connection of the SSA chamber later senses a ΔP similar to that of a cell stuck far from the progesterone source in the CH chamber. In both systems, spermatozoa are stimulated by incremental values of ΔP, which tend to become constant at the end of the experimental period (Fig. 1 C, inset ii, and D). It is worth noting that ΔP curves in the 10 pM range are identical to those estimated for the 10 nM and 10 µM ranges (Fig. S1).

### Picomolar gradients of progesterone stimulate either the acrosome reaction or priming for the acrosome reaction in different sperm subpopulations

**Figure 2:**
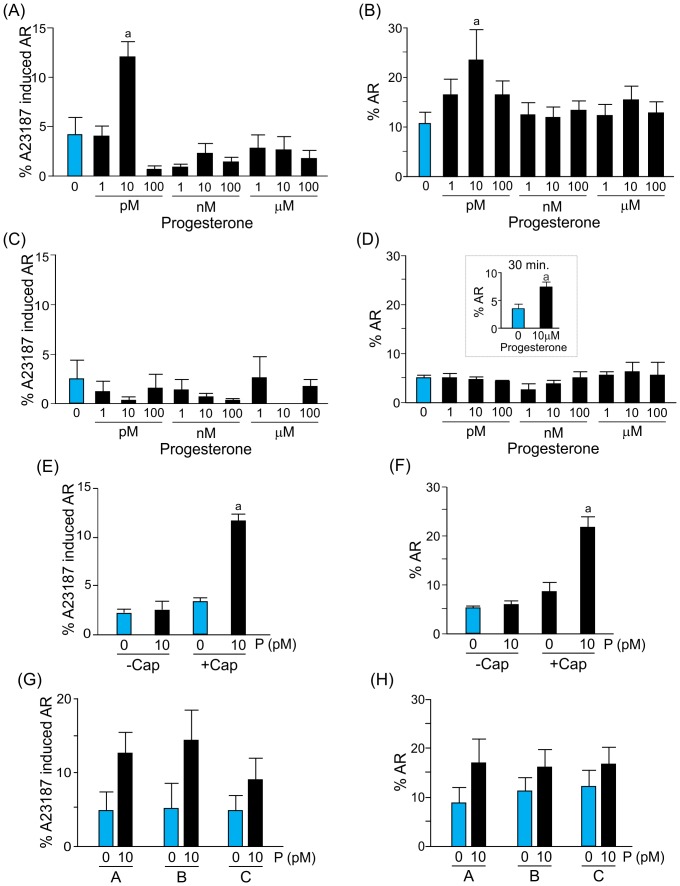
Picomolar gradients of progesterone generated in the chemotaxis chamber stimulate either priming or the acrosome reaction in different sperm subpopulations. Percentage of spermatozoa primed (A) or acrosome-reacted (B) after a 15 min exposure to gradients of different progesterone concentrations (1 pM to 100 µM). Percentage of spermatozoa-primed (C) or acrosome-reacted (D), after a 15 min exposure to a homogeneous distribution of different progesterone concentrations (1 pM to 100 µM). Data are shown as means ± s.e.m. calculated from 7 independent experiments, counting 200–600 cells per treatment. ^a^, significant differences vs. without progesterone (*p*<0.05). Inset: Percentage of acrosome-reacted spermatozoa after a 30 min exposure to a homogeneous 10 µM progesterone concentration. Data are shown as means ± s.e.m. calculated from 3 independent experiments, counting 200-600 cells per treatment. ^a^, significant differences vs. without progesterone (*p*<0.05). Percentage of spermatozoa-primed (E) or acrosome-reacted (F) in capacitated (+cap) or non-capacitated (-cap) populations, after a 15 min exposure to a gradient of 10 pM progesterone. Data are shown as means ± s.e.m. calculated from 4 independent experiments, counting 200–600 cells per treatment. ^a^, significant differences vs. without progesterone (*p*<0.05). Percentage of spermatozoa-primed (G) or acrosome-reacted (H) in the presence or absence of progesterone, located in different longitudinal areas of the connection between wells (A, close to the progesterone well; B, in the middle; and C, close to the culture medium well).

Capacitated spermatozoa adhered to a coverslip were exposed in the CH chamber to gradients generated from different concentrations of progesterone, ranging from 1 pM to 100 µM. The percentage of spermatozoa that were primed for the acrosome reaction was significantly higher when the cells were exposed to a 10 pM gradient of progesterone, compared to the cells kept in the absence of the steroid (Fig. 2A). Similarly, a significant increase was also observed in the percentage of spermatozoa that underwent the acrosome reaction upon stimulation with a 10 pM gradient of progesterone (Fig. 2B). These effects (AR and priming) were elicited by different sperm subpopulations, since the exposure to 10 pM progesterone induces the acrosome reaction in ∼14% of the sperm, whereas a subsequent stimulation with A23187 further increases the proportion of acrosome reacted cells by around 8% (Fig. 2A and B).

To verify whether these effects were due to the gradual distribution of the hormone, the same concentration of progesterone was homogeneously loaded in both wells and over the connection between them. None of the progesterone concentrations stimulated priming or the acrosome reaction (Fig. 2C and D). The acrosome reaction was elicited (Fig. 2D, inset) only with a step high concentration of progesterone (10 µM; six orders of magnitude higher than that effective as a gradient) and after 30 min of incubation (twice the time of the exposure to the gradient).

To test whether this dual effect of progesterone was dependent on capacitation, the level of spermatozoa that underwent the acrosome reaction or were primed for this by a gradient of 10 pM progesterone, was assessed in sperm previously incubated under capacitating conditions or immediately after seminal plasma removal in the absence of albumin. Both the induction of the acrosome reaction and the priming effect mediated by a 0–10 pM gradient of progesterone were observed only in spermatozoa previously incubated under capacitating conditions (Fig. 2E and F). Additionally, the priming and the acrosome reaction observed under a 0–10 pM gradient of progesterone seem to be independent of the sperm distance to the progesterone well (Fig. 2G and H).

**Figure 3:**
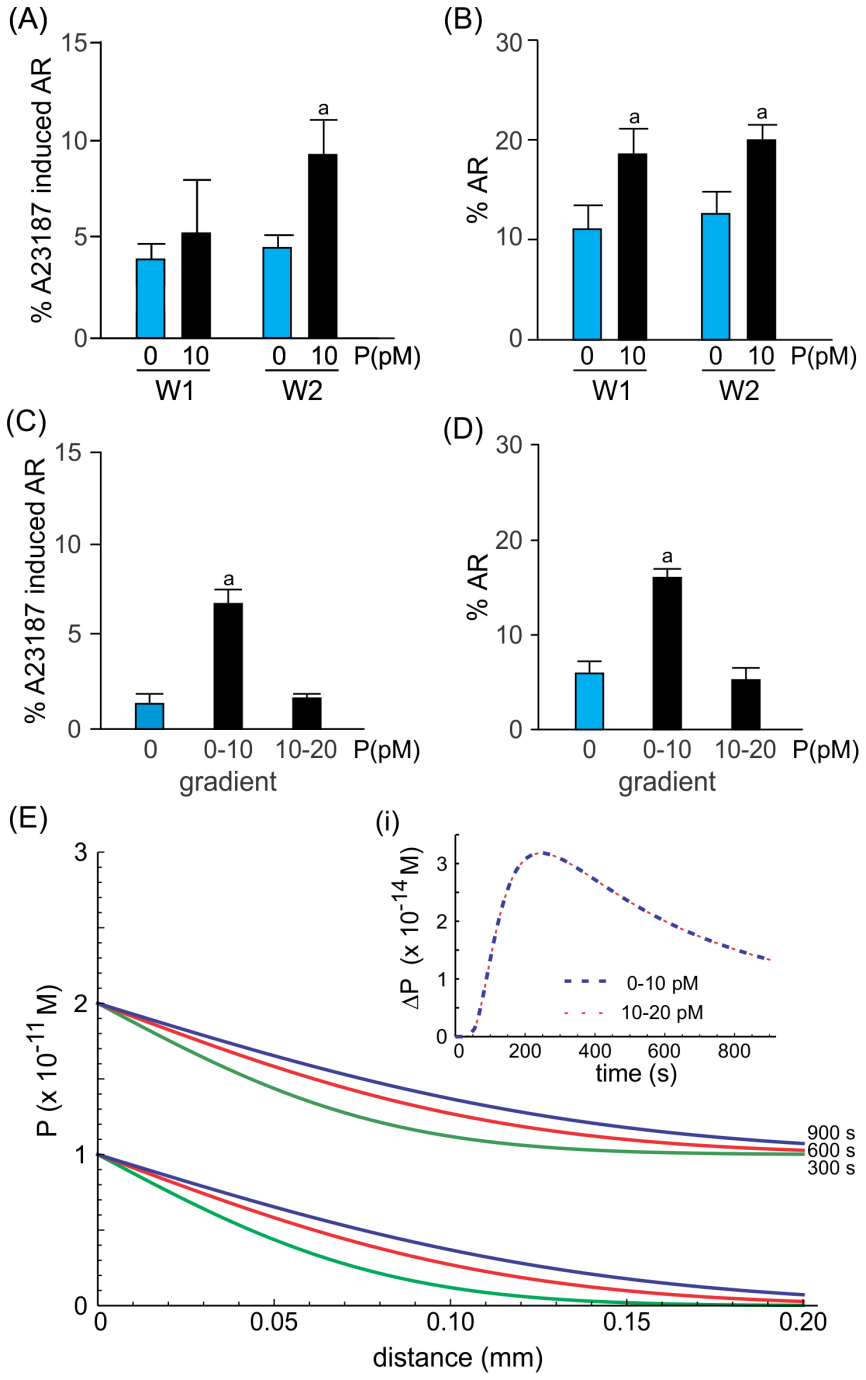
Picomolar gradients of progesterone generated in the SSA chamber stimulate either priming or the acrosome reaction in different sperm subpopulations. Percentage of spermatozoa primed (A) or acrosome-reacted (B) after a 20 min exposure to a gradient of 10 pM progesterone. Data are shown as means ± s.e.m. calculated from 5 independent experiments, counting 200–600 cells per well per treatment. ^a^, significant differences vs. without progesterone (*p*<0.05). Percentage of spermatozoa-primed (C) or acrosome-reacted (D) exposed to 0–10 pM or 10–20 pM of progesterone gradients. Data are shown as means ± s.e.m. calculated from 5 independent experiments, counting 200–600 cells per well per treatment. ^a^, significant differences vs. the other treatments (*p*<0.05). E, theoretical progesterone gradient formation over time and the connection between wells, when spermatozoa are exposed to 0–10 pM or 10–20 pM gradient of progesterone. In each set of curves, the lower represents time = 300 s, the middle = 600 s, and the upper = 900 s. The inset (i) represents the ΔP in the middle of the connection between both wells, for a 0–10 pM (blue dash line) and 10–20 pM (red dashed line) gradients.

The effect of a 0–10 pM gradient of progesterone on sperm priming and the acrosome reaction was also studied in the SSA chamber. The percentage of spermatozoa that were primed for the acrosome reaction was significantly increased only in the sperm population recovered from well 2 containing progesterone (Fig. 3A). The exposure to the progesterone gradient also stimulated the acrosome reaction in a sperm subpopulation isolated from well 2 (Fig. 3B). Surprisingly, a similar effect was also observed in the sperm population recovered from well 1 (Fig. 3B), suggesting that spermatozoa that underwent the acrosome reaction upon progesterone stimulation may be randomly distributed between wells. Since the priming and AR effect of progesterone was observed only when cells were exposed to 0–10 pM gradient of the steroid, we next tested whether the concentration or the ΔP was responsible for the observed effect. For that, sperm were exposed in the SSA to a progesterone gradient of 0–10 pM or 10–20 pM. As predicted by Fick's law, the ΔP values over time are the same for a 0–10 pM or 10–20 pM gradient of progesterone, only differing in the absolute concentration level of the steroid along the connection tube (Fig. 3E and inset i). Since the priming and AR effect were observed only under a 0–10 pM gradient of progesterone (Fig. 3C and D), this result suggests that the biological effects are dependent not only on the gradual distribution of progesterone but also on the steroid dose.

Regardless of the way spermatozoa are exposed to the progesterone gradient (fixed position or swimming), the dual effect of a picomolar gradient of progesterone was observed in different sperm subpopulations, with sizes for priming and acrosome reaction that were respectively: 7.8±1.5% and 13.5±6.5% (for CH chamber) and 4.8±1.1% and 7.0±2.3% (for the SSA chamber). Thus, in both systems, the level of sperm that underwent the acrosome reaction upon the progesterone stimulus was about 1.5 times that of the priming effect.

**Figure 4:**
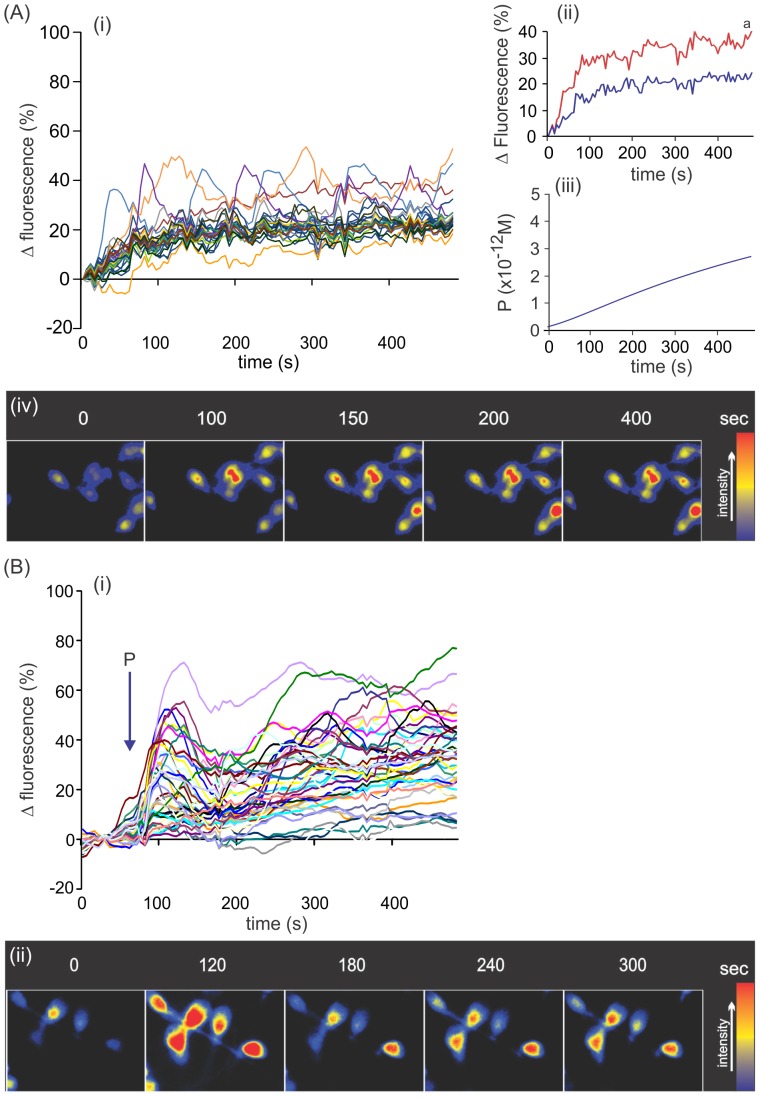
Picomolar gradients of progesterone stimulate singular intracellular calcium changes in spermatozoa. A, cells exposed to a gradient of 10; Aii, comparison of the curves showing mean values of the subpopulation of oscillating sperm with calcium values higher than the mean population value (red) and the mean of the rest of the cells (blue); Aiii, absolute progesterone concentration in the middle of the CH chamber along time where the starting point corresponds to 2 min due to the delay between CH sealing and start of recording. ^a^, significant differences vs. the blue curve (*p*<0.0001). B, cells exposed to a step application of 10 pM progesterone. Bi, calcium variations in several single cells along time. Representative images of several sperm cells exposed either to a gradient or a step 10 pM progesterone (Aiv and Bii, respectively). In Ai and Bi, data are shown as calcium variation expressed as relative fluorescence intensity (%) of individual cells of one representative experiment of five, analyzing 50 cells per treatment and experiment.

Sperm capacitation and the acrosome reaction are dependent on intracellular calcium mobilization [27], which may be also stimulated by progesterone [28]. We examined whether the way of administering 10 pM progesterone stimulates singular intracellular calcium changes in spermatozoa. Calcium variations were analyzed in sperm stuck to a coverslip, previously loaded with a fluorescent calcium indicator dye and then exposed to 10 pM progesterone supplied either as a gradient in the CH chamber or as a step in an imaging chamber. Although in both settings the intracellular calcium increased immediately after progesterone stimulation, the pattern of calcium increase was different. In all spermatozoa exposed to a gradient of 0–10 pM progesterone, the intracellular calcium moderately increased until reaching a plateau without observing a transient initial peak (Fig. 4Ai and Aiv). However, a subpopulation of cells (∼11%) showed oscillations at a level of calcium above the mean population value, which continued throughout recording (Fig. 4Aii). Interestingly, the amount of progesterone molecules gradually increased along the experimental time similarly to calcium increment (Fig. 4Aiii). In addition, in this system there was no relation between the intracellular calcium increment stimulated by the progesterone gradient and the cell orientation (toward progesterone well, culture medium well, or along the no gradient axes; Fig. S2). When sperm cells were exposed to a micromolar gradient of progesterone, the pattern of calcium increase was similar (Fig. S3A). In contrast, sperm exposed to step 10 pM progesterone increased intracellular calcium with a typical biphasic response, comprised of a fast, transient increase followed by a sustained level of calcium, where the cells were distributed in a wide range of calcium increments (Fig. 4Bi and Bii). In addition, the pattern was similar to that observed with micromolar step concentrations of progesterone (Fig. S3B). The corresponding controls for gradient and step (calcium signaling with culture medium without progesterone) are shown as Fig. S4A and B.

## Discussion

In biological systems, cells are exposed to molecules distributed as a gradient; this is the only mode of interaction between cells and molecules *in vivo*. Moreover, cells are usually exposed to multiple gradients of different molecules that constantly vary over time [1]. Thus, the cells may evolve to respond to gradients more than to step doses. Therefore, cellular studies based on homogenous molecular stimulus may not be representative of natural environments. Spermatozoa are not the exception, since they are permanently stimulated by different molecular gradients in either the male or female reproductive tract. In this study we focused on the effect of progesterone gradients on sperm functions such priming and the AR. We described the versatile action of a gradual distribution of very low doses of progesterone, which selectively stimulate priming and the acrosome reaction in different sperm subpopulations. These results sustain the notion that the effect that progesterone has on the spermatozoon depends on: 1) the way the hormone is administered, 2) the steroid concentration, and 3) the physiological status of the spermatozoon.

### Which are the features of the progesterone gradient in both systems (CH and SSA) that are relevant for the biological interpretation of the results?

It is clear that the biological effect is observed only when the sperm cells are exposed to a progesterone gradient of 0–10 pM, but why? The formation of the progesterone gradient follows Ficks' law of diffusion, whereby at any spatial point along the gradient the level of progesterone increases over time (see Fig. S1 in [12]). However, the rate of progesterone increase varies according to the place the cell is located along the connection between wells and the dynamic condition of the cells (stuck or swimming). For instance, in the CH chamber a cell positioned near the border of the well containing the steroid will receive a more dramatic ΔP than another placed near the well without progesterone. However, this initial high ΔP (affecting only cells closer to the progesterone well) may not be responsible for the biological effects, since the cells respond similarly along the connection between wells and independently of their orientation (Fig. 2G and H; Fig. S2, respectively). However, the biological response may be related to a low but sustained ΔP, as observed at later times in the CH chamber (Fig. 1C, inset ii). Conversely, in the SSA, the cells are swimming and so the ΔP varies according to the cell speed and the time of entering the connection between wells (Fig. 1D). It is probable that the biological effects take place also when the ΔP is more stabilized. Since the orientation of the cells does not seem to differentially affect the stimulus of the biological response, the classical notion of a “temporal gradient” (generally accepted to explain the sperm chemotactic response) is also plausible for the sperm processes studied here. Even though ΔP curve's shape in the 10 pM range is identical to those estimated for 10 nM and 10 µM ranges (Fig. S1), the priming and the AR response was observed only in the 10 pM range (Fig. 2A and B). Moreover, by preserving the 10 pM difference of progesterone between wells, the 10–20 pM gradient of the steroid was not as effective as that generated by a 0–10 pM concentration of the hormone to induce priming and AR (Fig. 3C and D). It is worth to be noticed that in the step application, progesterone can form an extremely rapid gradient while applying to the cells; however, the homogeneous distribution of the steroid is very rapid, whereas after reaching the steady state ΔP is equivalent to zero. On the contrary, in the CH chamber, where progesterone is slowly and gradually supplied (the gradient can be maintained for several hours), ΔP is higher than zero even after several minutes as observed in Fig. 1C, inset ii. Even though there may be a gradient of progesterone in both settings, the timing can make the difference between them. Therefore, the biological response seems to be due to a concentration gradient generated by a specific progesterone concentration.

### Progesterone gradients as a natural way to stimulate sperm function

When sperm cells reach the oviduct, they interact with its epithelium where they perform the capacitation process [29]. It has been pointed out that sperm capacitation is an essential event for fertilization since only capacitated spermatozoa are able of chemotactic swimming, traversing the cumulus oophorus, undergoing the induced acrosome reaction, binding to the zona pellucida, binding and fusion to the oocyte membrane [30].The last steps of sperm capacitation have been associated with molecular preparation to further undergo the induced acrosome reaction [31]–[34]. During acrosomal exocytosis, several hydrolytic enzymes are released to the extracellular space favoring the sperm traversing towards the oocyte surface [35]. For years, the zona pellucida has been pointed out as the natural place for the induction of the acrosome reaction [36]. However, it was recently observed in the mouse that those spermatozoa that fertilize the egg undergo the acrosome reaction far from the zona pellucida [20], [21], opening the possibility of other inducers of acrosomal exocytosis *in vivo*.

Progesterone is a hormone that is present in the oviductal microenvironment and has been reported to stimulate several sperm functions under *in vitro* conditions [6]. For instance, high micromolar doses of the steroid applied as a step for long incubation periods have been reported to stimulate sperm capacitation [37]–[41]. Some authors suggested that progesterone primes spermatozoa for the induced acrosome reaction [17], [18], or helps binding to the zona pellucida [42], [43]. There are also a number of publications that reported progesterone as an inducer of the acrosome reaction, while others did not observe this effect [6]. For instance, when human spermatozoa were exposed to micromolar step doses of progesterone for short periods (5–15 min), some authors observed an increase in the level of acrosome reacted sperm [44]–[48], while others did not observe any effect on acrosome reaction even after longer periods of incubation with the hormone [49]–[51]. Moreover, Sagare-Patil et al [52] reported that progesterone induced acrosome reaction is time and concentration dependent which, in any event, was elicited with step micromolar concentrations of the steroid after 30 min of incubation.

In general, these sperm effects have been observed with step micromolar doses of progesterone, a condition different from that *in vivo* where the spermatozoa may face an increasing concentration gradient of the steroid while approaching to the egg.

Here we tested whether spermatozoa are able to undergo priming and the acrosome reaction upon stimulation with a progesterone gradient. When spermatozoa are exposed to a gradient of 10 pM progesterone, either from a fixed position or while swimming across the gradient, one subpopulation of sperm are primed for the acrosome reaction and another is directly induced to undergo the acrosome reaction. These effects are dependent on previous incubation under capacitating conditions, sustaining the notion that the priming effect of progesterone may be considered as a late event of sperm capacitation. In contrast, homogeneous distribution of the steroid did not cause similar effects and, moreover, the induction of the acrosome reaction was only observed at a progesterone step dose six orders of magnitude higher and after longer periods of incubation. These results suggest a different action of progesterone depending on the steroid administration and concentration.

Either the subpopulation of sperm that undergo priming or AR under the exposure to a 10 pM gradient of progesterone may be related to the subpopulation of cells that augmented the calcium with oscillations. However, our experimental conditions do not enable verification of the association between any of the biological sperm effects and the calcium oscillating cells. Nevertheless, we previously showed that the stimulation of sperm chemotaxis (also in a sperm subpopulation) by pM gradients of progesterone provoked intracellular calcium mobilization; the latter is a consequence of either calcium entry from extracellular space or exit from calcium intracellular stores [8]. Therefore, it would not be surprising that similar effects were also related to the priming and AR stimulated by a pM gradient of progesterone; however, further studies are needed to verify this hypothesis. In this study we reported calcium signaling upon pM progesterone (as a gradient and step), which are similar to those showed by others ([53] and [54], respectively). By using a system to generate a progesterone gradient, Harper et al [53] observed a significant calcium increment after 20 sec of initiation of progesterone gradient when the steroid concentration is probably extremely low, since nM concentration is reached after 3 min. Even though in both studies most of the cells showed significant calcium signaling during the first minute of exposure to progesterone, the latency to get the plateau is higher in Harper's study. This may be due to the fact that in their system the progesterone concentration continued rising until micromolar concentrations, whereas in ours the maximum level of progesterone reached is picomolar. Although their study differs from ours in several ways (the system to generate progesterone gradients, the experimental design and the conditions to determine calcium uptake), both studies showed additional similar results. For instance, the majority of the cells increased calcium upon progesterone stimulation (96% in their study vs. 100% in ours); the transient calcium increase was not observed; calcium oscillations once established, continued throughout recording; the latter were observed in a subpopulation of cells whereas their magnitude was similar even at higher concentrations of the steroid. Although a micromolar gradient of progesterone showed a similar pattern of calcium to that observed under a 10 pM steroid gradient, the priming and AR were not stimulated by this high steroid concentration gradient which may be related to other sperm functions as pointed out by others. Thus, sperm calcium oscillations upon micromolar progesterone gradient stimulation have been previously described which were more related to an increased flagellar beating (resemble to hyperactivation) but not to AR [53]. In addition, we observed a calcium biphasic response upon pM step progesterone that was similar to that previously reported (see figure S3 in [54]) which was also slightly lower than that observed at micromolar progesterone concentrations.

The calcium voltage channel Catsper has been recently reported to be stimulated with progesterone step doses from 0.1 nM to μM, suggesting that the sperm functions mediated by the steroid may be regulated by this calcium channel [55], [56]. Unfortunately, there is no experimental evidence that Catsper can be stimulated with a 10 pM gradient of progesterone. Interestingly, the inhibition of Catsper with voltage-gated calcium channels blockers mainly suppressed the transient response of calcium uptake stimulated by step progesterone [52], [56]. In ours and Harper's progesterone gradient system [53], extremely low concentrations of the steroid did not stimulate the unique classical transient calcium increase usually observed with step progesterone. Therefore, it is probable that the calcium signaling observed with a pM progesterone gradient may be independent of Catsper channel. Park [54] have already proposed that the transient calcium influx may be due to the CatSper calcium channel whereas the later sustained calcium signal may be mediated by a progesterone receptor, which is transferred to sperm by prostasomes, together with additional calcium signaling tools during ejaculation. In addition, when mibefradil was used at concentrations expected to inhibit Catsper, the acrosome reaction was partially inhibited [52], [57], [58], suggesting that the action of progesterone could be mediated also by other sperm molecular components as recently proposed [54], [59], [60]. The issue demands further investigations.

As a whole, the present results support the notion that the effect of progesterone on sperm function depends not only on the sperm's physiological state and the concentration of the steroid, but also on the way the steroid is administered to the cell.

### What might the biological significance be of the versatile action of negligible gradients of progesterone?

Previously in our lab we reported a chemotactic effect of a 10 pM gradient of progesterone [5], [8], [9], [12]. It is surprising that the same picomolar gradient of the hormone can induce such diverse phenomena in the sperm population as chemotaxis, priming for the acrosome reaction and induction of the acrosome reaction. One explanation would be the physiological heterogeneity of the sperm sample whereby different sperm subpopulations co-exist at any given time. Sperm capacitation is a transient phenomenon that is experienced by different sperm subpopulations over time [13]. Thus, after seminal plasma removal, few cells are transiently capacitated for few hours, and thereafter they become irreversibly postcapacitated, a status that may prevent sperm fertilizing the egg [13], [14]. These two distinctive features of capacitation, transience and asynchronicity, lead to a heterogeneous sample containing spermatozoa in different physiological states.

Our results are in line with the notion of sample heterogeneity mentioned above, since we observed spermatozoa at different physiological statuses: non-capacitated sperm (those that do not respond to the inducer for the acrosome reaction, either progesterone or calcium ionophore); sperm that are at the final steps of capacitation and are susceptible to be primed for the acrosome reaction (those that after the exposure to progesterone are able to acrosome react upon stimulation with calcium ionophore); spermatozoa that have completed the capacitation process (those that acrosome react after calcium ionophore stimulus without previous stimulation of progesterone). However, the physiological state of those spermatozoa that undergo the acrosome reaction under the induction of progesterone may have several interpretations. One possibility would be that these spermatozoa are postcapacitated, in which case progesterone negatively selects them by stimulating the acrosome reaction, as suggested by others [61], [62]. Another interpretation would be that these cells are fully ready to fertilize the egg, in agreement with recent results that suggest that fertilizing spermatozoa undergo the acrosome reaction far from the zona pellucida [20]. A third option would be that the progesterone-induced acrosome-reacted sperm subpopulation is comprised of fully capacitated and postcapacitated sperm. The latter possibility seems reasonable, given that the level of progesterone-induced acrosome-reacted sperm population is 1.5 times higher than that susceptible to be primed by progesterone. Thus, the physiological heterogeneity of the sperm population would be of biological importance.

In humans, in whom ovulation is periodic, the entrance of spermatozoa to the oviduct does not necessarily coincide with the presence of an egg. Therefore, if all the sperm population were to simultaneously capacitate themselves, the amount of sperm ready to fertilize would be depleted in a few hours. The biological strategy that counteracts this extreme situation would be the gradual provision of small “doses” of capacitated spermatozoa during longer periods, as previously suggested [13], [63], combined with the optimizing effect of picomolar gradients of progesterone based on sperm heterogeneity. Thus the probability that a spermatozoon at an optimum physiological state meets and fertilizes an egg inside the oviduct is increased and would be extended over time. Where a picomolar gradient of progesterone would be located *in vivo*? Although this question is currently difficult to answer due to the unavailability of appropriate techniques, such information would help to better interpret these results in the context of the physiological environment of the oviduct at the time of ovulation.

## Supporting Information

Figure S1 **Progesterone rate of change (ΔP) calculated in the middle of the connection between wells for 0–10 pM (blue line), 0–10 nM (red line) and 0–10 µM (green line) progesterone gradients.**
(TIF)

Figure S2 **Percentage of oscillating sperm with calcium values higher than the mean population value with the head oriented towards: the progesterone well (1), the opposite well containing culture medium (3), and up (2) or down (4) the no gradient axes.**
(TIF)

Figure S3 **Intracellular calcium increase in spermatozoa exposed to micromolar progesterone concentration supplied as a gradient (A) or step (B).** Data is shown as a representative experiment of 5.
(TIF)

Figure S4 **Intracellular calcium variations in sperm cell under control conditions in the CH chamber (A) or the imaging chamber (B).** Data is shown as one representative experiment of at least 3 independent experiments.
(TIF)
