# Supplementary figures and images for: Versatile Action of Picomolar Gradients of Progesterone on Different Sperm Subpopulations
Source: PLoS One. 2014 Mar 10;9(3):e91181. doi: 10.1371/journal.pone.0091181 (PMC3948779; doi:10.1371/journal.pone.0091181)

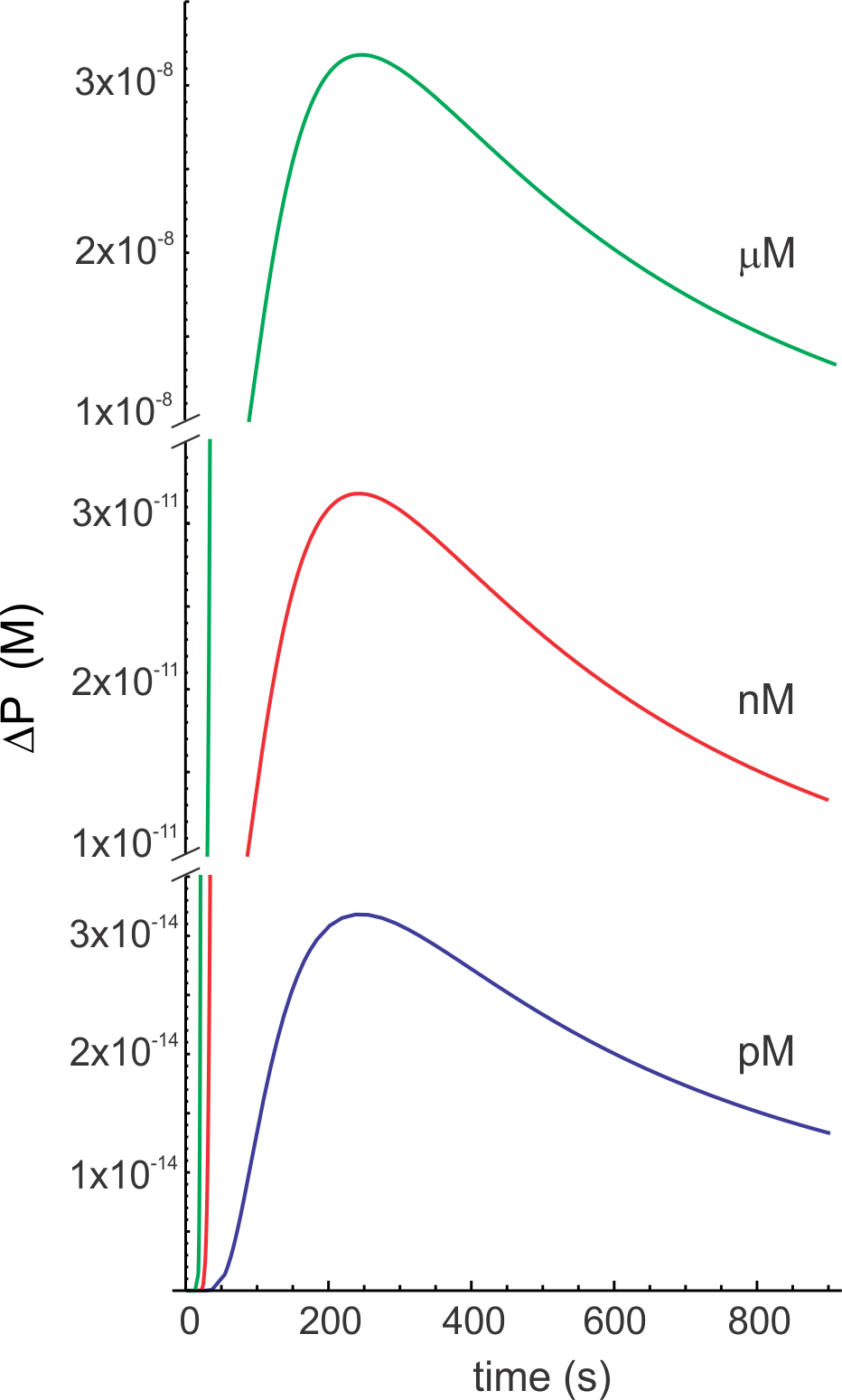

Supplement: Figure S1 — Progesterone rate of change (ΔP) calculated in the middle of the connection between wells for 0–10 pM (blue line), 0–10 nM (red line) and 0–10 µM (green line) progesterone gradients. (TIF) [file pone.0091181.s001.tif]

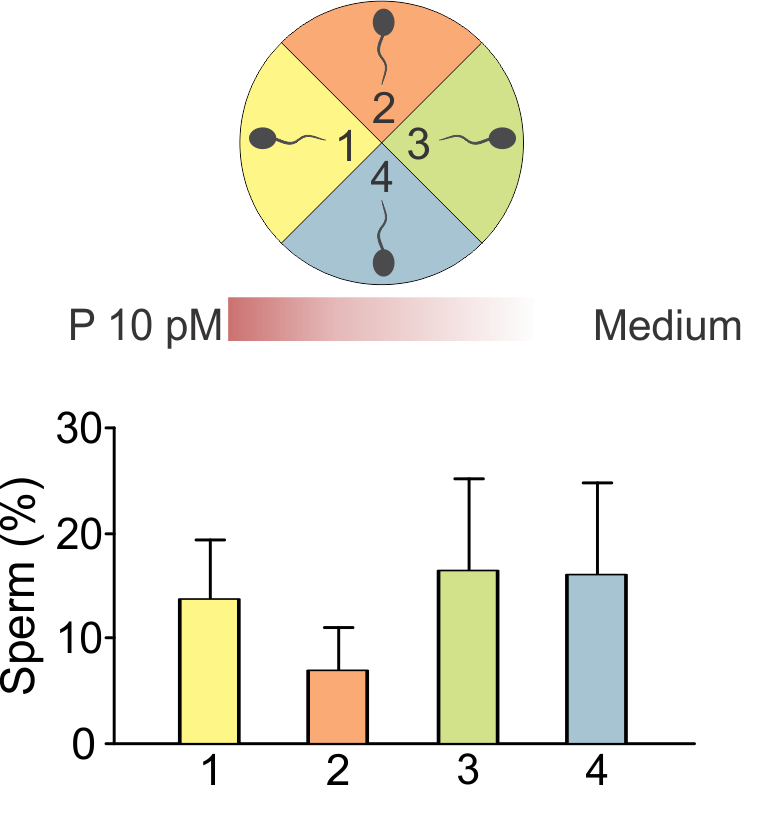

Supplement: Figure S2 — Percentage of oscillating sperm with calcium values higher than the mean population value with the head oriented towards: the progesterone well (1), the opposite well containing culture medium (3), and up (2) or down (4) the no gradient axes. (TIF) [file pone.0091181.s002.tif]

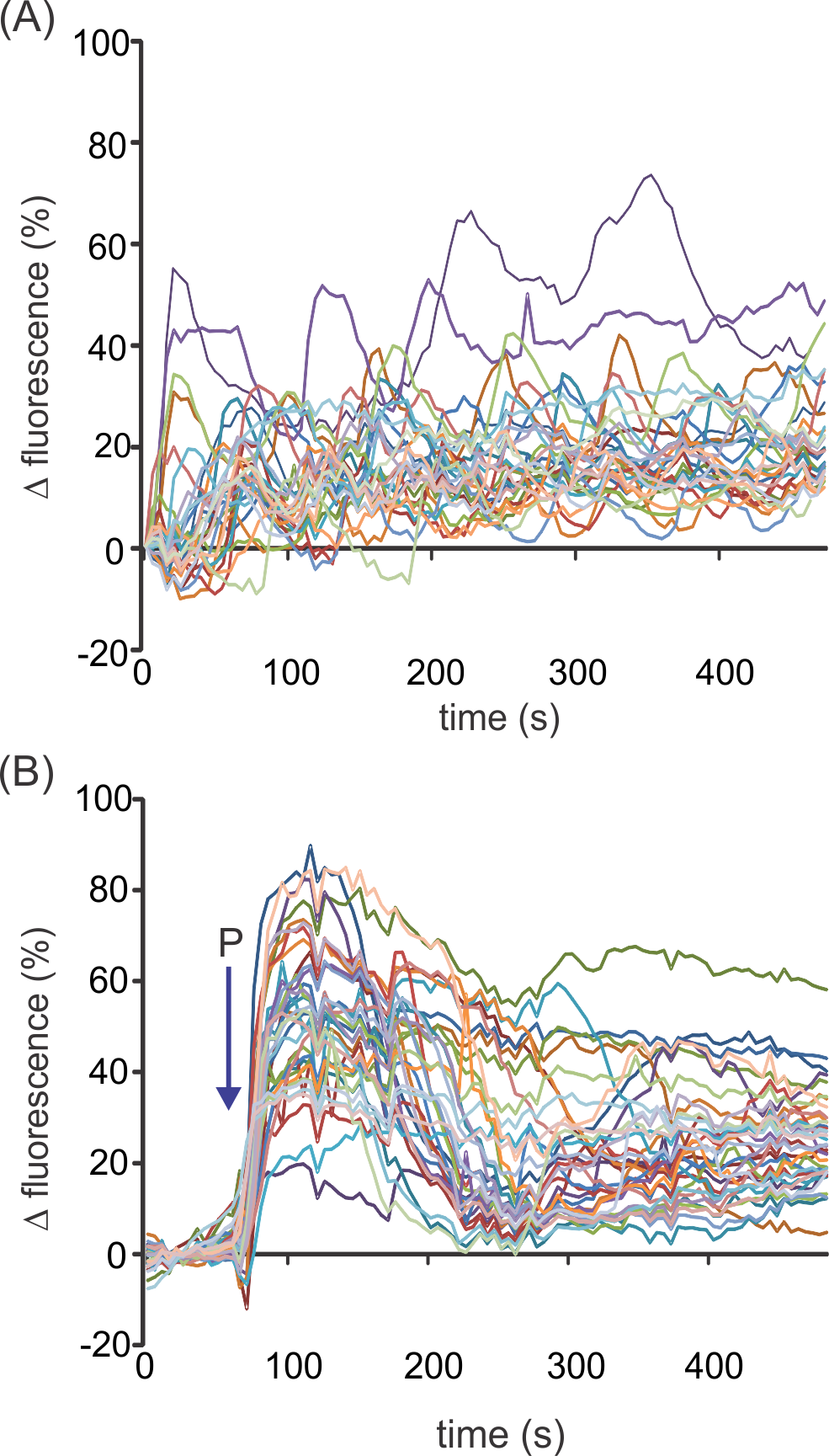

Supplement: Figure S3 — Intracellular calcium increase in spermatozoa exposed to micromolar progesterone concentration supplied as a gradient (A) or step (B). Data is shown as a representative experiment of 5. (TIF) [file pone.0091181.s003.tif]

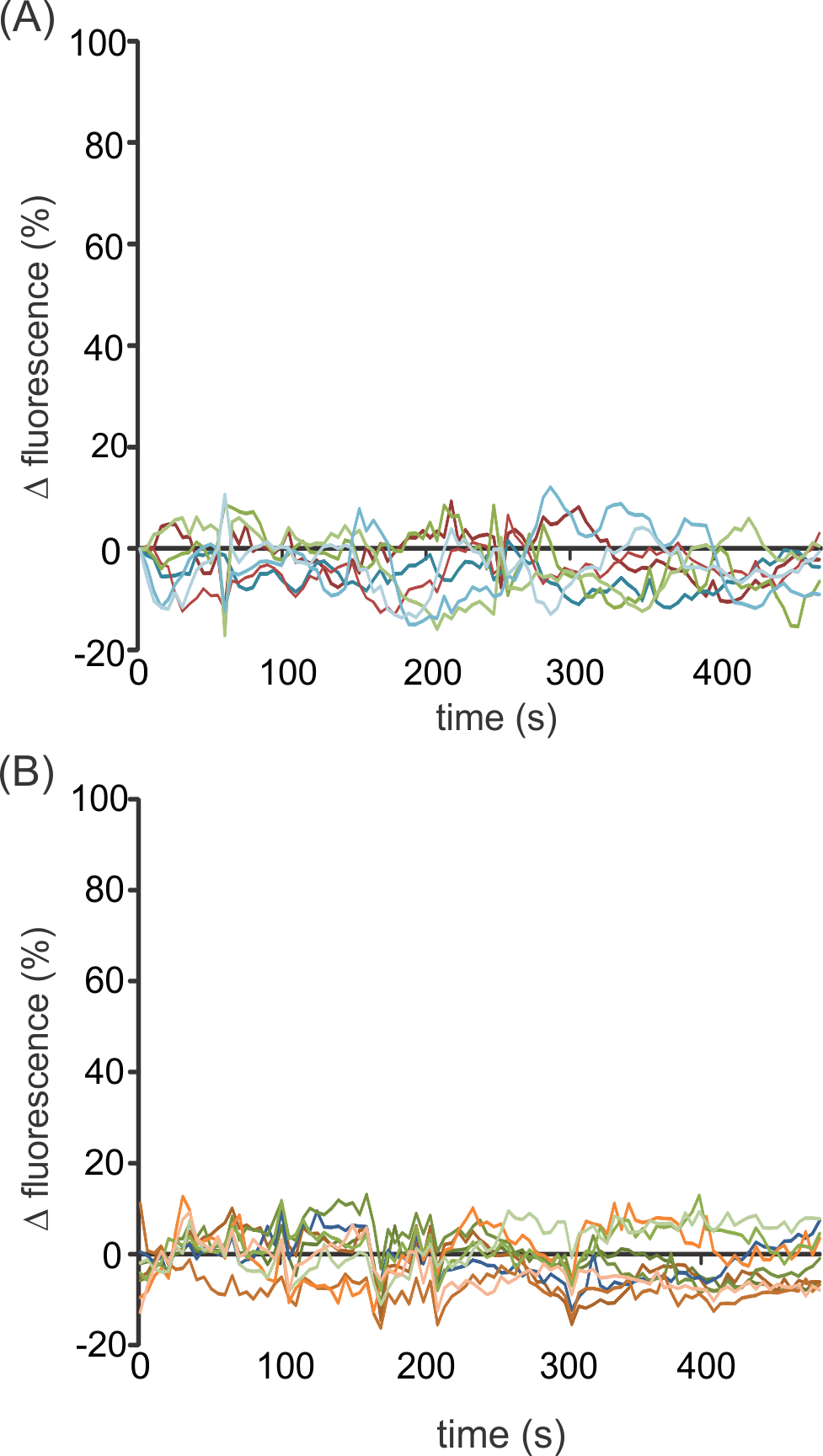

Supplement: Figure S4 — Intracellular calcium variations in sperm cell under control conditions in the CH chamber (A) or the imaging chamber (B). Data is shown as one representative experiment of at least 3 independent experiments. (TIF) [file pone.0091181.s004.tif]
